# MexS mediated heteroresistance of *Pseudomonas aeruginosa* to ciprofloxacin

**DOI:** 10.3389/fmicb.2026.1761186

**Published:** 2026-02-19

**Authors:** Xiaodan Ma, Yana Chen, Chenlu Xiao, Congcong Li, Lizhong Han, Chengxi Li, Jinjing Ni

**Affiliations:** 1 Anhui Key Laboratory of Infection and Immunity, Bengbu Medical University, Bengbu, Anhui, China; 2 Department of Biochemistry and Molecular Biology, Bengbu Medical University, Bengbu, Anhui, China; 3 Division of Life Sciences and Medicine, Department of Pediatrics, The First Affiliated Hospital of USTC, University of Science and Technology of China, Hefei, Anhui, China; 4 Department of Laboratory Medicine, Ruijin Hospital Affiliated to Shanghai Jiao Tong University School of Medicine, Shanghai, China; 5 Department of Clinical Microbiology, Ruijin Hospital Affiliated to Shanghai Jiao Tong University School of Medicine, Shanghai, China; 6 Laboratory of Bacterial Pathogenesis, Shanghai Institute of Immunology, Shanghai Jiao Tong University School of Medicine, Shanghai, China; 7 Department of Microbiology, Bengbu Medical University, Bengbu, Anhui, China

**Keywords:** ciprofloxacin heteroresistance, mexS, *P. aeruginosa*, pilus-mediated virulence-associated functions, twitching

## Abstract

Bacterial heteroresistance has been increasingly identified as an important phenomenon for many antibiotic/bacterium combinations. However the mechanisms underlying ciprofloxacin heteroresistance in *P. aeruginosa*, a key drug for treating this pathogen, remain poorly understood. In this study, 11 ciprofloxacin-heteroresistant *P. aeruginosa* isolates were identified by screening 226 clinical strains. Whole-genome sequencing (WGS) identified recurrent mutations within the *mexS* gene among these isolates. Crucially, CRISPR-Cas9-mediated deletion of *mexS* in the representative heteroresistant isolate (7318HR) abolished the heteroresistance phenotype, thereby confirming its functional necessity. Transcriptomic profiling following *mexS* deletion identified 532 differentially expressed genes involved in multiple biological processes, including phenazine biosynthesis, propanoate metabolism, bacterial secretion systems, and quorum sensing. Notably, genes associated with type IVb-tad pili were significantly downregulated. Consistent with this, the *mexS* mutant exhibited concurrent impairments in virulence-associated traits, including markedly reduced twitching motility (by approximately 38%) and polystyrene adhesion capacity (by approximately 50%) compared to the wild-type strain. These findings demonstrate that *mexS* is a key genetic determinant of ciprofloxacin heteroresistance in *P. aeruginosa* and concurrently modulates pilus-mediated virulence-associated functions.

## Introduction

*Pseudomonas aeruginosa* is an aerobic, gram-negative bacterium characterized by a single polar flagellum. It is a major opportunistic pathogen responsible for nosocomial infections (Holmes et al., 2021; Krell and Matilla, 2024) and causes substantial mortality annually, particularly in cases involving antibiotic-resistant strains (Holmes et al., 2021). Ubiquitous in the environment, *P. aeruginosa* exhibits intrinsic resistance mechanisms that allow it to evade most conventional antibiotics (Pang et al., 2019). Multidrug-resistant variants account for a considerable proportion of clinical isolates, significantly complicating treatment (Botelho et al., 2019; Qin et al., 2022). In recognition of its threat, the World Health Organization designated *P. aeruginosa* as a high-priority pathogen in 2024 (World Health Organization [WHO], 2024). Studies in recent years have reported the presence of heteroresistance in clinical settings that have been associated with treatment failure. Heteroresistance describes a phenotype in which a subpopulation of bacterial cells exhibits a higher level of antibiotic resistance than the predominant susceptible population (Andersson et al., 2019).

The current criteria for defining bacterial heteroresistance require that the minimum inhibitory concentration (MIC) of the resistant subpopulation be at least eight-fold higher than that of the susceptible main subpopulation, and that the frequency of this resistant subpopulation reaches or exceeds 10^−7^ (Andersson et al., 2019; Luo et al., 2024). These stringent criteria, coupled with the reliance of routine clinical microbiology laboratories on methods (such as standard broth microdilution or disk diffusion) that may not detect minority resistant subpopulations, contribute to the underdetection of heteroresistance in clinical practice. Identified mechanisms underlying heteroresistance include—but are not limited to—spontaneous tandem amplification of resistance genes (Andersson et al., 2019; Dewachter et al., 2019), upregulation of drug efflux pump genes and downregulation of porin proteins (He et al., 2018; Xu Y. et al., 2020), and the activity of two-component regulatory systems (Doijad et al., 2023; Kang et al., 2019). Additionally, essential genes involved in DNA repair, as well as quorum sensing genes *lasI* and *rhlI*, have also been implicated in this phenotype (Li et al., 2022; Lu et al., 2022).

Although heterogeneous resistance in *P. aeruginosa* to various antibiotics has been extensively studied (Egge et al., 2024; Howard-Anderson et al., 2022; Jia et al., 2020), its underlying mechanisms remain incompletely elucidated, particularly in the context of ciprofloxacin for *P. aeruginosa* infections. Furthermore, the specific prevalence and clinical impact of ciprofloxacin heteroresistance may be underestimated due to the aforementioned detection challenges. In this study, we identified 11 clinical isolates of *P. aeruginosa* exhibiting heteroresistance to ciprofloxacin. Utilizing high-throughput sequencing, we were screened for key genes involved in this phenotype and identified *mexS* as a critical determinant. The biological functions of *mexS* were further investigated to clarify its role in heteroresistance.

## Results

### Screening of ciprofloxacin heteroresistance isolating strains

**FIGURE 1:**
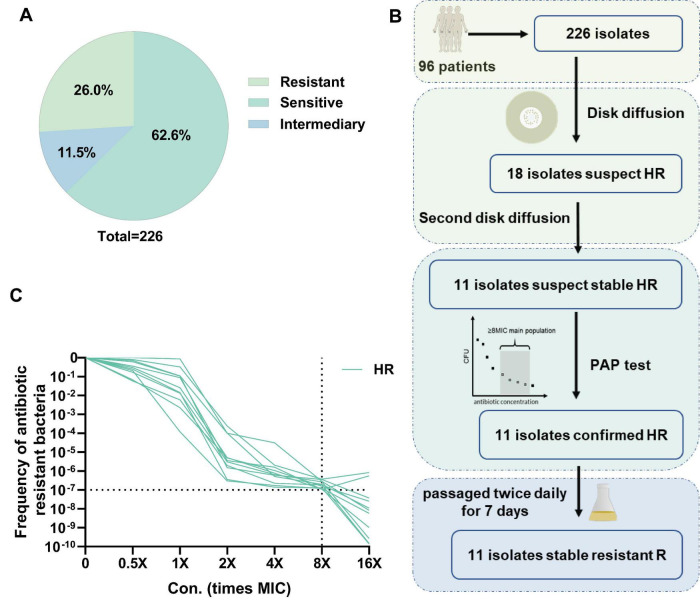
Detection of heteroresistance and drug sensitivity to ciprofloxacin in clinical isolates of *Pseudomonas aeruginosa*. **(A)** Statistical analysis of MIC test of 226 clinical isolates to ciprofloxacin. **(B)** Screening workflow and main results of heteroresistant isolates (HR). **(C)** PAP (population analysis profiling) of HR isolates.

A total of 226 *P. aeruginosa* isolates collected from sputum, alveolar lavage fluid, drainage fluid and midstream urine samples from 96 patients in Ruijin and Renji hospitals (Ethics statement: The study received ethical approval from the Ethics Committee of the Ruijin Hospital, Shanghai Jiao Tong University (No. 2023-79). Informed consent was obtained from all participants) were tested for heteroresistance. Most of these patients are critically ill, with a small number from patients with chronic infections such as COPD and bronchiectasis. Firstly, we evaluated 226 isolates for drug resistance to ciprofloxacin using the Clinical and Laboratory Standards Institute (CLSI) operational methods for antimicrobial susceptibility testing. Among these isolates, 26 (11.5%) were intermediate, 59 (26.11%) were resistant, and 141 (62.39%) were susceptible (Figure 1A). We found some monoclonal colonies within the inhibition zones of some isolates (Supplementary Figure 1). In order to identify the potentially heteroresistant isolates, we conducted two rounds of screening by the disc diffusion method and identified 11 suspected isolates. For clarity, the primary clinical isolates exhibiting this phenotype are hereafter referred to as heteroresistant isolates (HR). We further validated the 11 candidate HR isolates by PAP testing. To obtain HR isolates that could generate stable resistant subpopulations (R), we further selected monoclones from the inhibitory region of antimicrobial susceptibility disk and tested them for ciprofloxacin resistance after 14 consecutive passages on antibiotic-free plates. Each passage involved transferring a 1:100 dilution of the culture to fresh medium and incubating for 12 h. The R subpopulations derived from these 11 HR isolates are indicated in Figure 1B. The PAP curve indicated the frequency of the resistant subpopulation of PAO1 at 4-fold MIC has reached 10^–10^, which was much lower than 10^–7^, while the frequency of the 11 HR isolates at 8-fold MIC was still higher than 10^–7^ (Figure 1C).

To further determine whether the resistant strains isolated from the HR isolates still retained the heteroresistance characteristics, we also conducted a PAP test on the R subpopulations, and the results are shown in Supplementary Figure 2, where all the R subpopulations obtained by isolation from the HR isolates lost the heteroresistance features. Nevertheless, the MIC of ciprofloxacin was determined by R subpopulations and HR isolates, and it was found that the MIC of R subpopulations was generally more than 8-fold elevated compared to that of HR isolates, and the results are shown in Table 1.

**TABLE 1:** Susceptibilities of heteroresistant pseudomonas aeruginosa isolates (HR) and their derived resistant subpopulations (R).

HR Isolate	MIC (mg/L)	R subpopulation	MIC (mg/L)	MIC fold change (R/HR)
30HR	0.5	30R	4	8
1595HR	8	1595R	64	8
6655HR	0.125	6655R	4	32
7314HR	0.06	7314R	1	16.7
7318HR	0.125	7318R	2	16
7500HR	0.125	7500R	2	16
7637HR	0.06	7637R	1	16.7
7755HR	0.06	7755R	1	16.7
7866HR	0.5	7866R	4	8
7895HR	0.06	7895R	2	33.3
A601HR	0.125	A601R	1	8

### Genome sequencing analysis of HR isolates and R subpopulations

**FIGURE 2:**
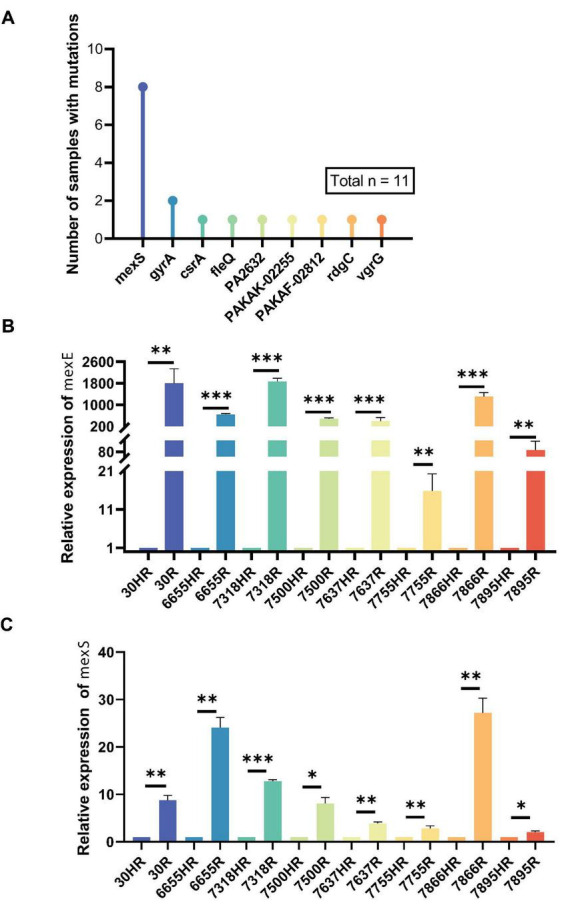
The proportion of strains with genetic mutations between R isolates selected from HR isolates and their corresponding parental heteroresistant strains, along with the transcription levels of the efflux pump-associated genes *mexE* and *mexS*. **(A)** The number of strains with genetic mutations when comparing R strains with their corresponding HR strains. **(B)** RT-qPCR analysis of *mexE* gene expression in resistant subpopulations (R) carrying *mexS* mutations cultured in LB medium, with expression levels normalized to 1 relative to the corresponding heteroresistant isolates (HR). **(C)** RT-qPCR analysis of *mexS* gene expression in resistant subpopulations (R) carrying *mexS* mutations cultured in LB medium, with expression levels normalized to 1 relative to the corresponding heteroresistant isolates (HR). *Represents *p* ≤ 0.05, **represents *p* ≤ 0.01, ***represents *p* ≤ 0.001 indicating significant differences between the R subpopulations and the parental HR strains. RT- qPCR, real-time quantitative reverse transcription PCR.

Since the R subpopulations lost their heteroresistance relative to their parental HR isolates, we performed whole-genome sequencing of the 11 HR isolates and their corresponding subpopulations. Analysis of the genetic variants in the R subpopulations revealed that single nucleotide variants (SNVs) were the predominant type (Figure 2A). Notably, mutations in the *mexS* gene were identified in 8 of the 11 pairs, and mutations in *gyrA* were found in 2 pairs. Other genes, including *csrA*, *fleQ*, *PA2632*, *PAKAK-02255*, *PAKAF-02812*, *rgdC*, and *vgrG*, each harbored a mutation in a single isolate.

Given the high prevalence of *mexS* mutations (8/11), we focused our subsequent analysis on this gene. Among these eight HR-R pairs, three R subpopulations (30R, 7550R, and 7637R) harbored deletions in *mexS*, with 30R and 7550R containing larger deletions of 11 and 15 nucleotides, respectively. One R subpopulation (6655R) contained a single-nucleotide insertion. These insertion/deletion events all resulted in frameshift mutations. The remaining four R subpopulations (7318R, 7755R, 7860R, and 7895R) contained single-nucleotide substitutions, leading to either missense mutations or a premature stop codon (nonsense mutation). The specific *mexS* mutations identified in the R subpopulations relative to their paired HR isolates are detailed in Table 2, which provides the nucleotide positions, sequence changes, and predicted amino acid consequences. The MIC of ciprofloxacin in the R strains with the *mexS* mutation and their corresponding HR isolates was examined, and it was found that all R subpopulations exhibited a significant increase in MIC relative to the HR isolates, with an 8–33.33-fold increase in MIC (Table 1). It has been demonstrated that the inactivation of MexS can result in the overexpression of the drug efflux pump *mexEF-oprN* (Xu C. et al., 2020), which in turn leads to an increased resistance to antibiotics. The transcript levels of *mexS* and the efflux pump genes *mexE* were examined by RT-qPCR between the eight R isolates with *mexS* mutations and their parents. The results demonstrated that the relative transcript levels of *mexE* were significantly higher in the R subpopulations than those of the HR isolates (Figure 2B). A similar trend was observed for *mexS* (Figure 2C).

**TABLE 2:** *mexS* gene mutations identified in heteroresistant (HR) *Pseudomonas aeruginosa* isolates and their derived resistant (R) subpopulations.

Strain (ID)	Genotype (HR vs. R)	Contig	Nucleotid e position	Reference	Alter	Amino acid change	Mutation type
30	30HR/30R	NODE_10	29,007	CACCCGGCCACG	C	p. (AA330-333fs)	Frameshift
6655	6655HR/6655R	NODE_1	30,008	G	GA	p. (AA118fs)	Frameshift
7318	7318HR/7318R	NODE_1	904,371	T	C	p. (Phe259Ser)	Missense
7550	7550HR/7550R	NODE_2	1,700	GCCGACTCGTTGCCGC	G	p. (AA225-247fs)	Frameshift
7637	7637HR/7637R	NODE_18	26,539	CG	C	p. (AA142fs)	Frameshift
7755	7755HR/7755R	NODE_7	1,239	C	A	p. (Trp42Leu)	Missense
7860	7860HR/7860R	NODE_2	925	G	A	p. (Gln147stop)	Nonsense
7895	7895HR/7895R	NODE_1	2,002,359	T	C	p. (Leu46Pro)	Missense

The table lists mutations identified by comparing the genome sequences of the heteroresistant parental isolate (HR) with that of its derived stable resistant subpopulation (R) for each strain. “Nucleotide Position” is based on the assembled contig of the HR isolate. “Amino Acid Change” is predicted from the mutation in the R subpopulation relative to the HR sequence.

### Characterization of the genetic mechanism of heteroresistance to ciprofloxacin

**FIGURE 3:**
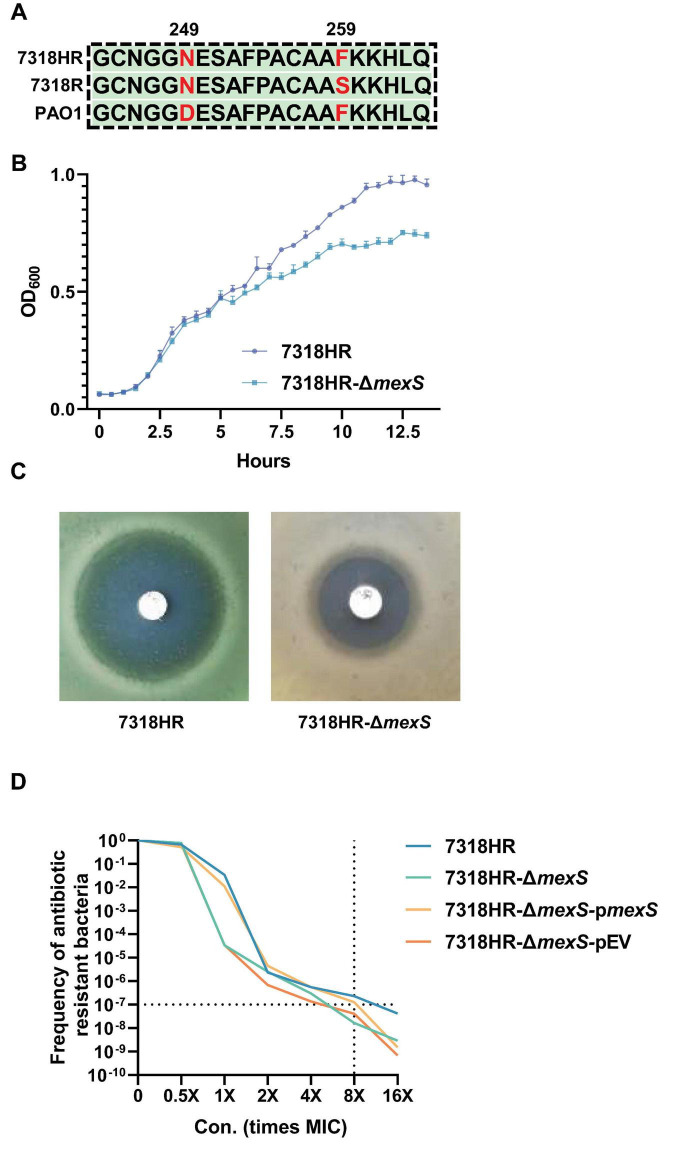
Sequence comparison of MexS among strains 7318HR, 7318R, and PAO1, as well as the impact of *mexS* gene deletion on the growth of strain 7318HR and its resistance to ciprofloxacin. **(A)** Sequence comparison of MexS among strains 7318HR, 7318R, and PAO1. **(B)** The growth curves of isolate 7318HR and its deletion mutant in LB medium at 37°C. **(C)** Susceptibility results of isolate 7318HR and its deletion mutant to ciprofloxacin on MH agar plates. **(D)** PAP curve of isolate 7318HR, 7318HR-Δ*mexS*, and 7318HR-Δ*mexS*-p*mexS*.

To further elucidate the correlation between *mexS* and heteroresistance, the sequence of MexS in HR isolates was initially examined. It was ascertained that all HR isolates exhibited an identical MexS sequence. A comparison of the MexS sequence with that of the laboratory strain PAO1 revealed a single amino acid difference, namely, the 249th amino acid, which was asparagine (N) for HR isolates and aspartic acid (D) for PAO1 (Figure 3A). Subsequently, we employed the CRISPR/Cas9 system to induce a knockout of the *mexS* gene in the 7318HR strain. By measuring the growth curves of strains 7318HR-Δ*mexS* and 7318HR in LB medium without antibiotics, it was found that the stationary-phase biomass of 7318HR-Δ*mexS* was approximately 20% lower than that of 7318HR (Figure 3B).

The results demonstrated that the *mexS*-deficient strains exhibited a loss of the heteroresistance phenotype to ciprofloxacin (Figure 3C). This was evidenced by the absence of single colonies in the zone of inhibition and the incompatibility of the PAP test with heteroresistance, as illustrated in Figure 3D. In parallel, the complementation of *mexS*-deficient strains by plasmids carrying *mexS* sequences from 7318HR isolates resulted in the reversion of heteroresistance phenotypes, thereby confirming the association of the *mexS* gene with heteroresistance to ciprofloxacin in *P. aeruginosa*. This also illustrates the intricacy of the heteroresistance mechanism to ciprofloxacin in *P. aeruginosa*, which does not facilitate the transformation of non-heteroresistant strains into heteroresistant strains through a single expression of the *mexS* gene.

### RNA sequencing analysis of *mexS* deletion mutants

Given the *mexS* mutant phenotypes observed across all HR isolates and the suitability of this strain for subsequent studies, we selected 7318HR to focus our investigations. In order to gain further insight into the function of the *mexS* gene in *P. aeruginosa*, we conducted a transcriptome sequencing analysis of three strains: 7318HR, 7318HR-Δ*mexS*, and 7318R. This analysis was performed under culture conditions without antibiotics, and the number of genes whose expression was detected in the transcriptomes of all three strains exceeded 6,000.

**FIGURE 4:**
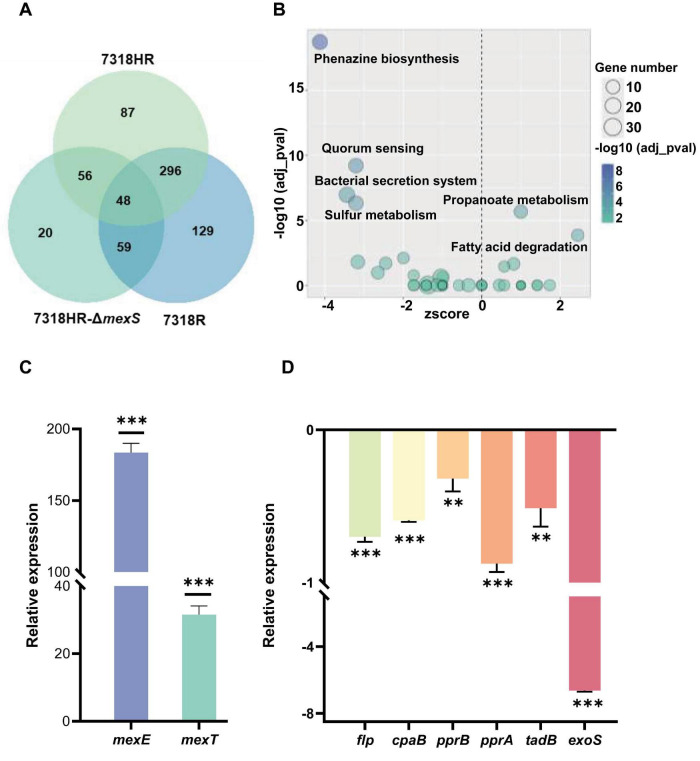
Transcriptome analysis of isolate 7318HR, 7318R, and 7318HR-Δ*mexS*, and qPCR quantification of efflux pump genes and flagellar assembly genes. **(A)** Venn diagram of differentially expressed genes (DEGs) in transcriptomes of isolate 7318HR, 7318R, and 7318HR-Δ*mexS*. Each circle contains the set of differentially expressed genes (DEGs) derived from the comparison of that strain against the other two. The “7318HR” circle contains DEGs from the comparison of 7318HR against 7318HR-Δ*mexS*. The “7318R” circle contains DEGs from the comparison of 7318R against 7318HR. The “7318HR-Δ*mexS*” circle contains DEGs from the comparison of 7318HR-Δ*mexS* against 7318R. **(B)** KEGG pathway enrichment analysis of differentially expressed genes between 7318HR and 7318HR-Δ*mexS*. **(C)** RT-qPCR analysis comparing *mexE* and *mexT* gene expression between 7318HR and 7318HR-Δ*mexS* cultured in LB medium, normalized using expression levels in 738HR. Positive values after normalization indicate upregulation of the respective gene in the mutant strain relative to 7318HR. **(D)** RT-qPCR analysis was performed to compare expression levels of flagellar synthesis genes (*flp*, *cpaB*, *pprB*, *pprA*, *tadB*) and the virulence gene *exoS* in isolate 7318HR and 7318HR-Δ*mexS* cultured in LB broth. Data were normalized against isolate 738HR, where negative values indicate downregulation of the respective gene in the Δ*mexS* mutant relative to 7318HR. * Represents *p* ≤ 0.05, **represents *p* ≤ 0.01, ***represents *p* ≤ 0.001 indicating significant differences between the R subpopulations and the parental HR strains. RT- qPCR, real-time quantitative reverse transcription PCR.

A differential expression analysis of the transcriptomes of the three strains revealed that, in comparison to 7318HR, strains 7318R and 7318HR-Δ*mexS* exhibited a higher number of differentially expressed genes. The number of differentially expressed genes was 532 and 487, respectively, whereas strains 7318R and 7318HR-Δ*mexS* exhibited differential expression of only 183 genes. The Venn plots of the number of differentially expressed genes (DEGs) for the three strains are presented in Figure 4A. The data indicate that the DEGs were primarily concentrated in the two analyzed groups, 7318R-vs.-7318HR and 7318HR-Δ*mexS*-vs.-7318HR. In contrast, the expression of the genes in the 7318R-vs.-7318HR-Δ*mexS* group exhibited a relatively limited range of variation. The 7318R and 7318HR-Δ*mexS* strains exhibited a relatively close transcriptional profile (Supplementary Figure 3), suggesting that the majority of genes exhibiting altered transcriptional levels in 7318R were associated with the occurrence of a mutation in *mexS*.

The DEGs in 7318HR-Δ*mexS*-vs.-7318HR were analyzed in order to identify the pathways that were most significantly enriched by KEGG database. The top five pathways identified were Phenazine biosynthesis, Propanoate metabolism, Bacterial secretion system, Quorum sensing, and Sulfur metabolism. In addition, deletion of *mexS* also affected energy metabolism and the two-component system of *P. aeruginosa*, as shown in Figure 4B. In the bacterial secretion system-enriched pathway, we observed that deletion of *mexS* caused a down-regulation of transcript levels of type III secretion system (T3SS)- and type VI secretion system (T6SS)-related genes, suggesting that *mexS* also plays an important role in infection, colonization and virulence of *P. aeruginosa*.

Transcriptional analysis via RT-qPCR revealed significant upregulation of multidrug efflux genes (*mexE*, *mexT*) and downregulation of flagellar biosynthesis associated genes in *P. aeruginosa* 7318HR*-ΔmexS* mutant compared to 7318HR strain. Specifically, *mexE* and *mexT* exhibited 183- and 31-fold increases, respectively (Figure 4C), while flagellum biogenesis genes displayed 32–87% reductions (Figure 4D), consistent with RNA-seq expression profiles.

### Effect of *mexS* on type IVb-tad pili assembly and associated phenotypes

Transcriptome analysis revealed that deletion of the *mexS* gene led to significant downregulation of genes encoding type IVb-tad pili. This finding was confirmed by RT-qPCR analysis of key pilus-related genes, including *flp*, *cpaB*, *pprB*, *pprA*, and *tadB* (Figure 4D).

**FIGURE 5:**
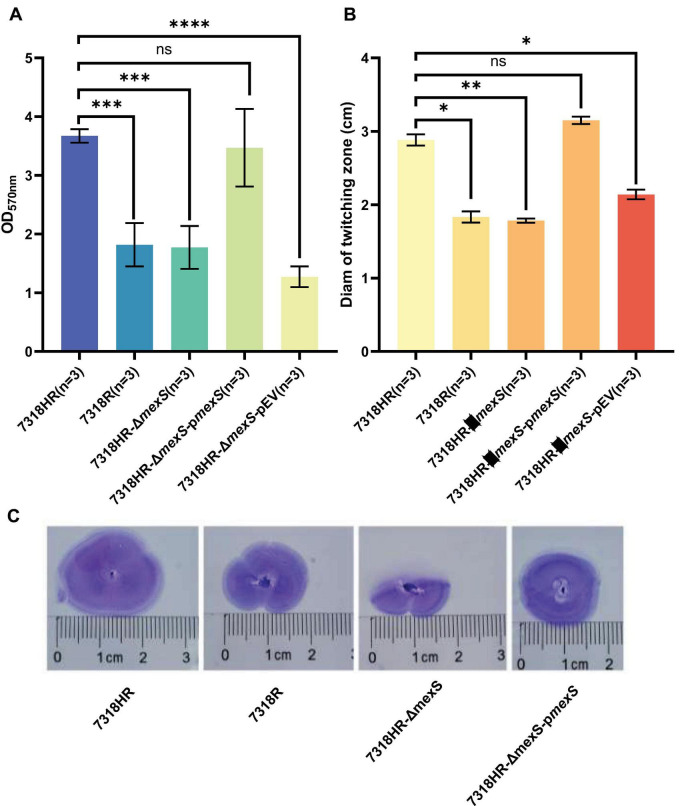
Impact of *mexS* deletion on *Pseudomonas aeruginosa* surface attachment to inert substrates and twitching motility. **(A)** Surface attachment assessment of isolate 7318HR, 7318R, 7318HR-Δ*mexS*, 7318HR-Δ*mexS*-p*mexS* and 7318HR-Δ*mexS*-pEV on abiotic surfaces. LB medium was added to sterile 24-well polystyrene plates. Overnight bacterial cultures were diluted 1:100 into the plates, followed by static incubation at 37°C overnight. After aspirating the culture, 1% (w/v) crystal violet solution was added for 5 min staining. The plates were decolorized with 95% ethanol, washed, and finally added in 600 μL ultrapure water for OD_570_ measurement. The data for each isolate is derived from three independent biological replicate experiments. ****p < 0.0001, ***p < 0.001, ns, not significant, two-tailed *t*-test. **(B)** Assessment of twitching motility capability for strains 7318HR, 7318R, 7318HR-Δ*mexS*, 7318HR-Δ*mexS*-p*mexS*, and 7318HR-Δ*mexS*-pEV on LB agar plates. Single colonies of each strain were stab-inoculated into 3-mm-thick 1% LB agar plates. After 16-h incubation at 37°C, plates were stained with 1% (w/v) crystal violet. Following removal of unbound dye by washing, diameters of twitching motility zones were measured. The data for each isolate is derived from three independent biological replicate experiments. **p < 0.01, *p < 0.05, ns, not significant, two-tailed *t*-test. **(C)** Crystal violet staining results of twitching motility zones for strains 7318HR, 7318R, 7318HR-Δ*mexS*, 7318HR-Δ*mexS*-p*mexS*, and 7318HR-Δ*mexS*-pEV on LB agar plates. Staining procedure was identical to that described for panel **(B)**.

Given the established role of type IVb-tad pili in twitching motility and surface adhesion in *P. aeruginosa*, we hypothesized that *mexS* deletion would impair these functions. Phenotypic assays supported this hypothesis: the Δ*mexS* mutant exhibited a significant reduction in both polystyrene adhesion capacity (approximately 50% decrease) (Figures 5A) and twitching motility (approximately 38% decrease in zone diameter) (Figures 5B,C) compared to the wild-type strain 7318HR. Importantly, these defects were fully restored upon genetic complementation with a plasmid carrying *mexS* (Figures 5A–C), confirming the specific role of *mexS* in regulating these pilus-mediated traits.

## Discussion

### Clinical resistance prevalence and heteroresistance identification

A total of 226 *P. aeruginosa* strains isolated from 96 patients at Ruijin and Renji hospitals were analyzed for ciprofloxacin susceptibility. Among these, 59 strains (26.11%) exhibited resistance, indicating a notably high prevalence of clinical resistance. Further screening via disc diffusion and population profiling identified 11 isolates showing heteroresistance to ciprofloxacin.

### *mexS* as a key genetic determinant of heteroresistance in strain 7318HR

Whole-genome sequencing of heteroresistant isolates and their stably resistant counterparts revealed that mutations in the *mexS* gene underlie the heteroresistant phenotype to ciprofloxacin. Of particular note, in the representative model strain 7318HR, *mexS* is annotated as a putative quinone oxidoreductase. Previous studies indicate that inactivating mutations in *mexS* can activate the *mexEF*-*oprN* operon via the transcriptional regulator MexT (Sobel et al., 2005; Wang et al., 2024), resulting in efflux pump overexpression and enhanced drug resistance. Additionally, MexS has been shown to suppress the expression of T3SS-related genes in a MexT-independent manner (Jin et al., 2011). Sequence analysis revealed notable diversity at amino acid position 249 of MexS: it is asparagine (N) in strains PAK and PA14, and aspartic acid (D) in PAO1. All 11 heteroresistant isolates in this study carried MexS-N249, which represses MexT transcriptional activity, whereas MexS-D249 does not. In contrast, MexS-D249 inhibits T3SS independently of MexT, while MexS-N249 lacks this ability (Jin et al., 2011; Sobel et al., 2005). The specific genetic and phenotypic analyses that follow focus on elucidating the role of *mexS* within the context of strain 7318HR.

### Transcriptomic and phenotypic consequences of mexS deletion in 7318HR

Transcriptome analysis comparing the *mexS*-deficient mutant (7318HR-Δ*mexS*) with the wild-type (7318HR) strain showed that *mexS* deletion led to downregulation of T3SS and T6SS-associated genes in this strain. KEGG pathway enrichment analysis highlighted five significantly affected pathways: phenazine biosynthesis, propanoate metabolism, bacterial secretion system, quorum sensing, and sulfur metabolism. Except for propanoate metabolism, these pathways were generally downregulated. Moreover, *mexS* deletion influenced the expression of genes involved in energy metabolism, two-component systems, and type IV tad pili (Figure 5B). Although the number of differentially expressed genes was limited, their impact on metabolic synthesis in 7318HR was considerable. Phenotypic assays further demonstrated that *mexS* deletion 7318HR markedly impaired both twitching motility and surface attachment capability in this genetic background.

The transcriptomic changes observed upon *mexS* deletion are extensive, implicating diverse cellular processes. It is important to distinguish between the potential direct regulatory roles of MexS and the indirect, compensatory effects arising from global physiological rewiring. The strong downregulation of type III and type VI secretion system (T3SS/T6SS) genes aligns with previous reports of MexS influencing these pathways, potentially through mechanisms independent of its canonical regulator MexT (Jin et al., 2011). This suggests a more direct or proximal role for MexS in modulating virulence-associated secretion. Conversely, the broader alterations in metabolic pathways (e.g., phenazine biosynthesis, propanoate and sulfur metabolism) and two-component systems likely represent secondary, indirect consequences of the primary regulatory disruption. These changes may reflect the cell’s adaptive response to the metabolic perturbation caused by *mexS* loss and the consequent activation of the *mexEF-oprN* efflux pump, which is energetically costly. While our data robustly map the transcriptional landscape resulting from *mexS* deletion, definitive assignment of direct targets requires future complementary approaches, such as chromatin immunoprecipitation sequencing (ChIP-seq) for any identified DNA-binding regulators in this cascade or kinetic transcriptomics following *mexS* repression.

Clinical implications and study limitations in clinical practice, MIC is a commonly utilized method for assessing the susceptibility of pathogenic bacteria to a specific antibiotic (Nicoloff et al., 2019). The presence of heteroresistance strains is frequently misidentified as that of sensitive strains under conditions of low antibiotic concentrations. This results in the administration of antibiotic treatment that not only fails to cure the infection but also facilitates the screening of drug-resistant mutant strains (Jia et al., 2020). Consequently, the use of MIC data as the sole criterion for determining bacterial susceptibility to antibiotics presents a significant challenge. Therefore, the resolution of the heteroresistance mechanism of *P. aeruginosa* is of paramount clinical importance.

Collectively, our findings in the model strain 7318HR establish that inactivation of *mexS* is a critical factor underlying the ciprofloxacin heteroresistance phenotype observed in this clinical isolate. Nevertheless, several limitations should be noted. First, our detailed mechanistic investigation—including transcriptomics, CRISPR deletion, and in-depth phenotypic assays—was conducted primarily in a single, representative heteroresistant isolate, 7318HR. While this allows for a deep and controlled analysis within this strain, it necessarily limits the generalizability of the *mexS*-dependent mechanism to other clinical HR isolates without further validation. Second, even within strain 7318HR, the precise mechanistic link between *mexS* inactivation and the heteroresistance phenotype remains to be fully disentangled. The concomitant upregulation of the *mexEF-oprN* efflux pump suggests its involvement; however, whether the observed heteroresistance is solely attributable to efflux activation or stems from broader regulatory rewiring orchestrated by *mexS* warrants further investigation. Future studies employing efflux pump inhibitors and isogenic mutants lacking both *mexS* and *mexEF-oprN* will be crucial to delineate their individual contributions and to clarify if the mechanism underlying heteroresistance in this context is distinct from that of stable high-level resistance. Finally, given that heteroresistance is inherently a multifaceted trait, the potential synergistic effects between *mexS* and other resistance mechanisms, such as efflux pump overexpression or adaptive mutations in two-component regulatory systems, remain an essential avenue for future inquiry, particularly across a broader collection of clinical isolates.

## Materials and methods

### Strain activation and culture

A culture of *P. aeruginosa*, stored at −80°C, was prepared by streaking on lysogenic broth (LB) solidified with 1.5% agar and incubated at 37°C for 24 h. A single clone was picked and inoculated into 5 mL of LB liquid medium and incubated at 37°C with agitation at 250 rpm for 24 h. This bacterial culture was then used for subsequent experiments. Unless otherwise stated, LB medium was used throughout this study, and cultures were incubated at 37°C with shaking at 250 rpm.

### Disk-diffusion method

The disc diffusion assay was performed according to the protocol described in reference (Sherman et al., 2019). In brief, strains were activated on Mueller-Hinton (MH) medium. All other conditions and procedures were as described above. A sterile cotton swab dipped into the activated bacterial suspension and used to spread the suspension evenly on the MH solid medium. A sterile round paper disc containing 5 mg of ciprofloxacin was placed in the center of the solid medium. The incubator was set to 37°C for 48 h. The diameter of the inhibition zone was measured, and if distinct colonies were observed within the inhibition zone, the strain was preliminarily identified as exhibiting heteroresistance to ciprofloxacin.

### Population analysis profile test

The population analysis profile test was performed according to the methodology detailed in reference (Sherman et al., 2019). In brief, solid medium containing ciprofloxacin was prepared at concentrations corresponding to 0.5, 1, 2, 4, 8, and 16 times the minimum inhibitory concentration (MIC). The activated strains underwent serial 10-fold dilution to achieve gradients from 10^0^ to 10^–7^. Aliquots (100 μL) of each dilution were plated onto the antibiotic-containing media and incubated at 37°C for 48 h. Technical triplicates were performed for each condition. Post-incubation, colony enumeration was conducted, with mean colony counts calculated and visualized using GraphPad software.

### Determination of ciprofloxacin MIC

The minimum inhibitory concentration (MIC) determination was performed according to the protocol detailed in reference (Sherman et al., 2019). In brief, a ciprofloxacin solution was diluted with Mueller-Hinton (MH) liquid medium to a final concentration of 128 μg/mL. This solution was then sequentially two-fold dilutions to generate a gradient of antibiotic concentrations. Aliquots (100 μL) of each concentration were dispensed into sterile 96-well plates, with the final well of each row containing antibiotic-free MH medium as a negative control. The activated bacterial cultures were adjusted to an optical density at 600 nm (OD_600_) of 1.0 using MH liquid medium, followed by a 1,000-fold dilution. One hundred microliters of the diluted bacterial suspension were inoculated into the antibiotic-containing wells. After overnight incubation at 37°C, the OD_600_ value of each well was measured via a microplate reader. The MIC was defined as the lowest antibiotic concentration at which bacterial growth matched the control well.

### Heteroresistance stability assay

A monoclonal colony of the heteroresistant isolate in the vicinity of the small drug-containing paper disk in the disk diffusion assay (designated R1) was selected and subjected to serial passage on antibiotic-free LB plates. The strain was passaged twice daily (approximately every 12 h) for 7 days, with each passage involving the transfer of a 1:100 dilution of the culture to fresh antibiotic-free medium (resulting in a total of 14 passages and yielding R14). MICs of R1 and R14 were evaluated. In the event of no notable discrepancy, R14 was designated as the stable resistant subpopulation (R), while its parental strain was identified as the stable heteroresistant isolates (HR).

### Genome and transcriptome sequencing and analysis

#### Genome sequencing and mutation analysis

The activated bacterial suspension were transferred to fresh 5 mL LB liquid medium at a dilution of 1:100 and incubated at 37°C until the OD_600_ reached 1.0. At this point, the bacteria were collected by centrifugation. Bacterial genomic DNA was extracted using the Bacterial Genome Extraction Kit (TianGen, DP302-02). Using the Illumina platform and employing a paired-end sequencing strategy, the obtained raw data was filtered and genome sequences was assembled using the SPAdes software (Prjibelski et al., 2020). The Prokka software (Seemann, 2014) is employed for the purpose of predicting the gene set. Subsequently, we employed the BWA software (Li, 2013) for the comparison of reads from the second-generation sequencing of R strains with their counterparts on the HR genome. Finally, we employed the GATK software (Poplin et al., 2017) to identify the mutation sites between them.

#### RNA sequencing and transcriptomic analysis

For transcriptome analysis, biological triplicates (*n* = 3) of both the wild-type strain 7318HR and its isogenic Δ*mexS* mutant were processed independently. The activated bacterial suspension was transferred into fresh LB medium at a dilution of 1:100 and cultured until the OD600 reached a value between 1.0 and 1.5. Subsequently, 3 mL of the bacterial suspension was collected, and the extraction of total RNA from different strains was performed using the Bacterial RNA Extraction Kit (TianGen, DP430). RNA integrity was verified using an Agilent 2100 Bioanalyzer (RIN > 8.0).

The analysis workflow was as follows:

1. Raw sequencing reads were subjected to quality control using FastQC (v0.11.9) and trimmed for adapter sequences and low-quality bases using Trimmomatic (v0.39) (Bolger et al., 2014).
2. The cleaned reads were then mapped to the reference genome of strain 7318HR using HISAT2 (v2.2.1) (Kim et al., 2015).
3. Read counts for each annotated gene were generated using featureCounts (subread package v2.0.3) (Liao et al., 2014).
4. Differential gene expression analysis was performed using the R package DESeq2 (v1.34.0) (Love et al., 2014). The DESeq2 model internally normalized read counts using the median-of-ratios method. No batch effect correction was applied as all samples were processed in a single batch. The criteria for identifying differentially expressed genes (DEGs) were: |log2 (fold change) | ≥ 1 and an adjusted *p*-value (Benjamini-Hochberg method) ≤ 0.05.

The raw RNA-seq sequencing data have been deposited in the China National Center for Bioinformation (CNCB) under BioProject accession number PRJCA055673 (GSA: CRA037173, CRA037172).

### Construction of mutants

All genes knockout mutants were constructed using a genome editing method pCasPA/pACRISPR by combining the CRISPR/Cas9 and the phage λ-Red recombination systems (Chen et al., 2018). All plasmids were constructed using ClonExpress II One Step Cloning Kit (Vazyme).

### Quantitative real time-PCR

RNA was extracted in accordance with the aforementioned methodology. Following the isolation of total RNA, cDNA was synthesized from the extracted RNA using an RNA reverse transcription kit. cDNA was then quantified by fluorescence using a real-time fluorescence quantitative PCR kit. In this study, 16S rRNA was utilized as a reference, and the 2^–ΔΔCt^ value was calculated to indicate the relevant expression of the gene under investigation. The data were analyzed using GraphPad software. The primers utilized for the detection of all genes via qRT-PCR are enumerated in Supplementary Table 1.

### Twitching motility and bacterial inert surface adhesion test

This experiment was conducted in accordance with the methodology proposed by de Bentzmann et al. (2006). The procedure was as follows: 1. A single colony was selected from the plate of the activated strain, stabbed into the bottom of a 3 mm thick 1% LB agar plate, and incubated at 37°C for 16 h. The agar in the plate was then carefully taken out and stained with 1% crystal violet for 5 min. After washing away the excess dye, a clear twitching zone was visible, and the twitching motility of the strain was detected by measuring its diameter. The data for each isolation is derived from three independent biological replicate experiments.

For bacterial inert surface adhesion test, we employed the methodology proposed by Bieber et al. (1998). The procedure is as follows: select a monoclonal colony from the plate and inoculate it in 5 mL of LB liquid medium for an overnight incubation period. Next, 400 μL of LB liquid medium is to be added to a sterile polystyrene 24-well plate. The overnight bacteria are then transferred into the 24-well plate at a ratio of 1:100, and the plate is left at 37°C for an overnight culture. The bacterial fluid in the wells should be carefully aspirated and 400 μL of 1% crystal violet dye should be added for a period of 5 min. The excess dye should be removed by suction, followed by the addition of 400 μL of 95% ethanol. The solution should then be shaken thoroughly and mixed well. Subsequently, the ethanol should be aspirated and 600 μL of deionized water should be added. The degree of bacterial adhesion to the inert surface can be quantified by measuring the absorbance at 570 nm. The data for each isolation is derived from three independent biological replicate experiments.

### Ethics statement

The study received ethical approval from the Ethics Committee of the Ruijin Hospital, Shanghai Jiao Tong University (No. 2023-79). Informed consent was obtained from all participants. Sputum samples were collected from patients at Ruijin Hospital affiliated with Shanghai Jiao Tong University, and both the identities of the patients and their corresponding data included in the study were anonymized.

### Statistical analysis

Data from at least three independent biological replicates are presented as mean ± standard deviation (SD). Statistical analysis was performed using GraphPad Prism 9.0 software. Prior to comparative analysis, the normality of data distribution for each experimental group was assessed using the Shapiro-Wilk test. For comparisons between two groups: if data followed a normal distribution, an unpaired two-tailed Student’s *t*-test was applied; if not, the non-parametric two-tailed Mann-Whitney U test was used. A *p*-value of less than 0.05 was considered statistically significant.

## Data Availability

The whole-genome sequencing data for the heteroresistant (HR) Pseudomonas aeruginosa isolates generated in this study have been deposited in the NCBI Sequence Read Archive (SRA) under BioProject accession number PRJNA1038743. The genome sequencing data for the corresponding resistant (R) subpopulations and the transcriptomic (RNA-seq) datasets have been deposited to the China National Center for Bioinformation (CNCB) under BioProject accession number PRJCA055673 (GSA: CRA037173, CRA037172). All other data supporting the findings of this study are available from the corresponding author upon reasonable request.
