# Supplementary material for: MexS mediated heteroresistance of Pseudomonas aeruginosa to ciprofloxacin
Source: Front Microbiol. 2026 Feb 19;17:1761186. doi: 10.3389/fmicb.2026.1761186 (PMC12960592; doi:10.3389/fmicb.2026.1761186)
Supplement: Supplementary file 1 [file Data_Sheet_1.pdf]

Table S1 The primer names and sequences utilized in the present study

primers	sequences
spacer-mexS-F	GTGGcacccgcgcgccgagagcct
spacer-mexS-R	AAACaggctctcggcgcgcgggtg
HF-upstream-mexS-F	ttttgagatctgtccatacccatggTCTAGAtcatccaagaccgcccgctccgt
HF-upstream-mexS-R	atgcactgcagaggtttgcgggacgacccgcagcgaaaga
HF-downstream-mexS-F	Tctttcgctgcgggtcgtcccgcaaacctctgcagtgcat
HF-downstream-mexS-R	tctgaatggcgggagtatgaaaagtCTCGAGcgaactcgctggcgcgactcatg
check-mexS-F	aatagtcgtcgagggtcagc
check-mexS-R	cctaccgctacctgatccag
qFlp-132F	GAAGAACCTGACTCTGCTCGTG
qFlp-132R	GTGGGACTCAATACGGCAATC
qCpaB-120F	GTGATGCTCTTCGTGCGTGA
qCpaB-120R	CTGTCCATCGCTGCCCAC
qmexEF	aggtcgcctttcttcaccag
qmexER	acgagtgggacgaattcacc
qmexTF	gagaaactgttcctcggcca
qmexTR	aggtgggcgaagatttcctg
qCpaC-132F	ACGGCACCAACGACAACG
qCpaC-132R	CGCTGTAGTCCAGTTCGCTG
qPprB-166F	TCGCCGAATCGCTGAACT
qPprB-166R	TTGCCGAACAGCGGATTG
qPprA-237F	TGAGGAGTTGGGACCTGTGTT
qPprA-237R	GGAAACGACAGGTCAATGGC
qTadB-127F	GCTGATGCAGGCAGTGGA
qTadB-127R	AACCTCGTAGAGTTCGGCG
