# Supplementary figures and images for: MexS mediated heteroresistance of Pseudomonas aeruginosa to ciprofloxacin
Source: Front Microbiol. 2026 Feb 19;17:1761186. doi: 10.3389/fmicb.2026.1761186 (PMC12960592; doi:10.3389/fmicb.2026.1761186)

FigS1

PAO1

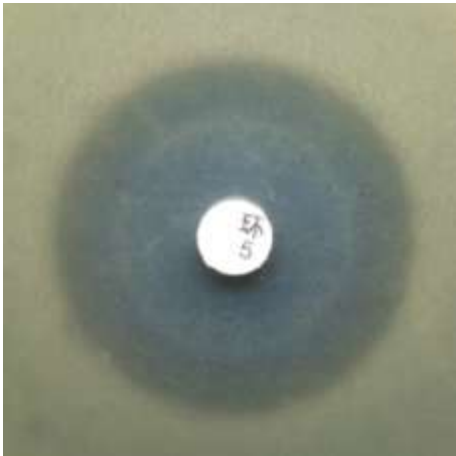

7318HR

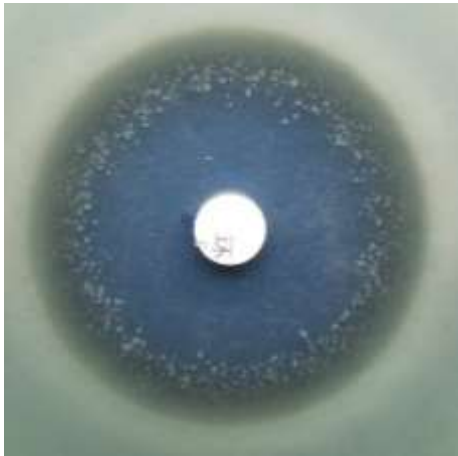

6655HR

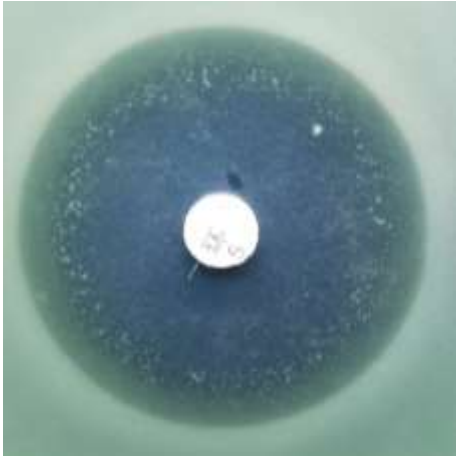

7500HR

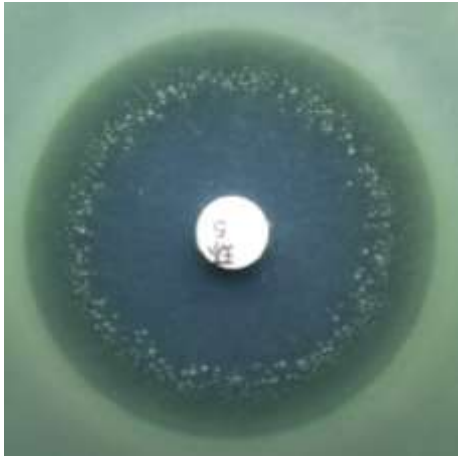

7637HR

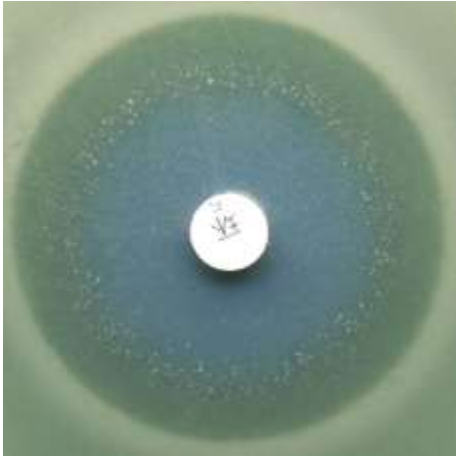

1595HR

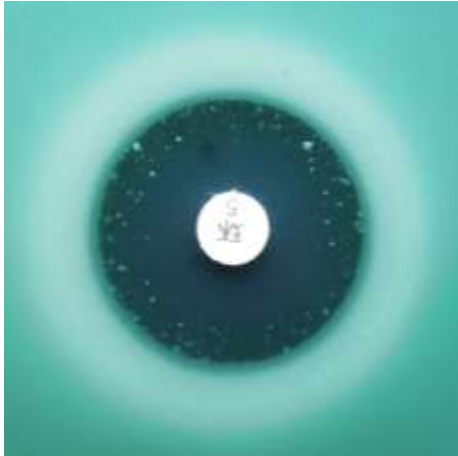

Supplement: Supplementary file 2 [file Image_1.pdf]

FigS2

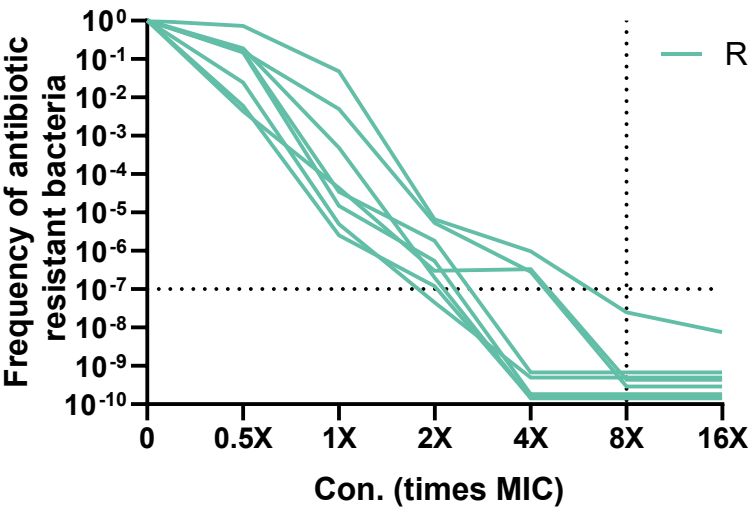

Supplement: Supplementary file 3 [file Image_2.pdf]

FigS3

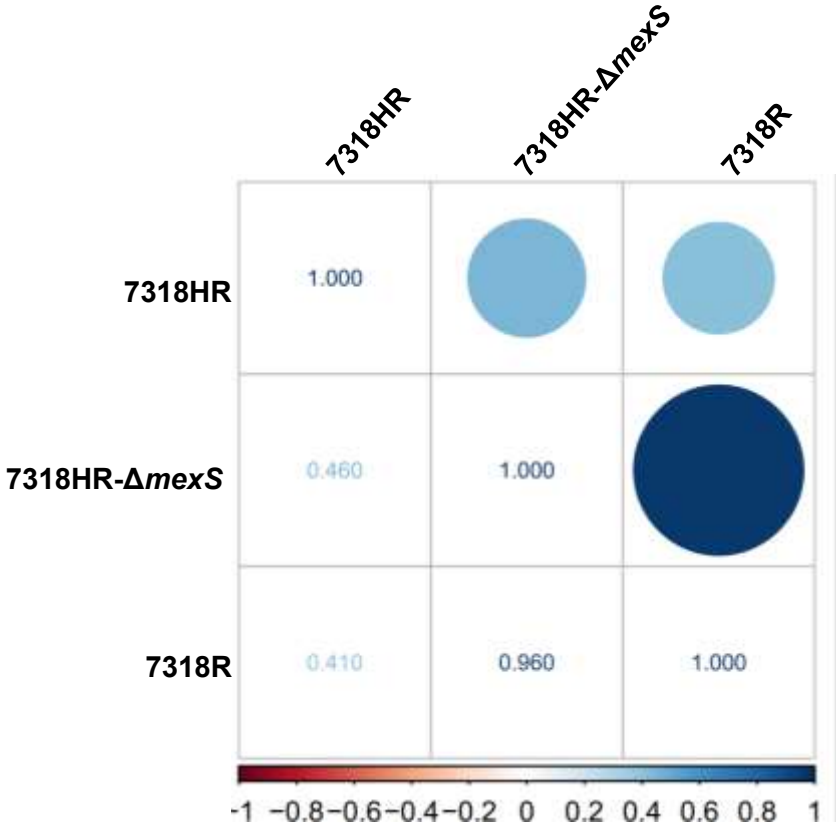

Supplement: Supplementary file 4 [file Image_3.pdf]
